# Poly[diaqua­bis­(μ-4,4′-bipyridine-κ^2^ *N*:*N*′)bis­(ethane-1,2-diol-κ*O*)bis(μ-sulfato-κ^2^ *O*:*O*′)dicobalt(II)]

**DOI:** 10.1107/S1600536813006685

**Published:** 2013-03-16

**Authors:** Kai-Long Zhong

**Affiliations:** a Department of Applied Chemistry, Nanjing College of Chemical Technology, Nanjing 210048, People’s Republic of China

## Abstract

In the title compound, [Co_2_(SO_4_)_2_(C_10_H_8_N_2_)_2_(C_2_H_6_O_2_)_2_(H_2_O)_2_]_*n*_, there are two crystallographically independent Co^II^ ions, each of which lies on a twofold rotation axis and has a slightly distorted octa­hedral environment. One Co^II^ ion is coordinated by two N atoms from two bridging 4,4′-bipyridine (4,4′-bipy) ligands, two O atoms from two sulfate ions and two O atoms from aqua ligands. The second Co^II^ ion is similar but with ethane-1,2-diol ligands in place of water mol­ecules. The sulfate anions act as bridging ligands to link two adjacent Co^II^ ions together, leading to the formation of linear ⋯Co1Co2Co1Co2⋯chains along the *a* axis. Adjacent chains are further bridged by 4,4′-bipy ligands, which are also located on the twofold rotation axis, resulting in a two-dimensional layered polymer extending parallel to (001). In the crystal, the layers are linked by extensive O—H⋯O hydrogen-bonding inter­actions involving the O atoms of the water mol­ecules and ethane-1,2-diol mol­ecules, resulting in a three-dimensional supra­molecular network.

## Related literature

For isostructural compounds, see: Zhong *et al.* (2011 ▶); Zhong (2013 ▶). For metal complexes with the 4,4′-bipyridine ligand, see: Tong & Chen (2000 ▶); Croitor *et al.* (2011 ▶); Lu *et al.* (2006 ▶, 1998 ▶); Luachan *et al.* (2007 ▶); Prior *et al.* (2011 ▶); Zhong & Qian (2012 ▶). For background to coordination polymers, see: Cui *et al.* (2002 ▶); Sarma *et al.* (2009 ▶); Zhang *et al.* (2010 ▶).
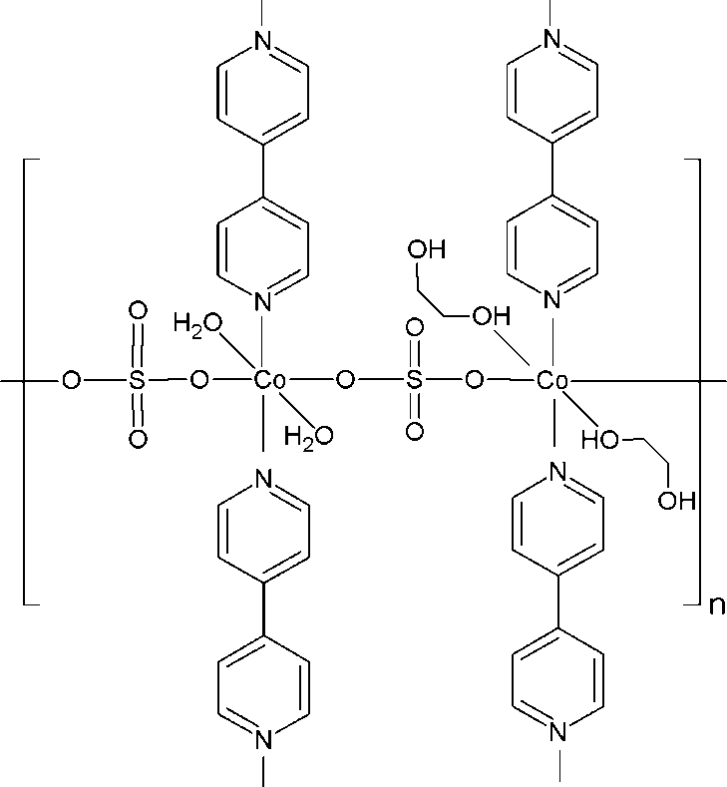


## Experimental

### 

#### Crystal data

[Co_2_(SO_4_)_2_(C_10_H_8_N_2_)_2_(C_2_H_6_O_2_)_2_(H_2_O)_2_]
*M* *_r_* = 782.53
Monoclinic, 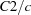

*a* = 11.124 (2) Å
*b* = 22.792 (5) Å
*c* = 12.066 (2) Å
β = 95.51 (3)°
*V* = 3045.2 (11) Å^3^
*Z* = 4
Mo *K*α radiation
μ = 1.30 mm^−1^
*T* = 223 K
0.35 × 0.25 × 0.20 mm

#### Data collection

Rigaku Mercury CCD diffractometer
Absorption correction: multi-scan (*REQAB*; Jacobson, 1998 ▶) *T*_min_ = 0.722, *T*_max_ = 1.000
8573 measured reflections
3453 independent reflections
3047 reflections with *I* > 2σ(*I*)
*R*_int_ = 0.020

#### Refinement

*R*[*F* ^2^ > 2σ(*F* ^2^)] = 0.029
*wR*(*F* ^2^) = 0.078
*S* = 1.05
3453 reflections
214 parameters
1 restraint
H-atom parameters constrained
Δρ_max_ = 0.44 e Å^−3^
Δρ_min_ = −0.49 e Å^−3^

### 

Data collection: *CrystalClear* (Rigaku, 2007 ▶); cell refinement: *CrystalClear*; data reduction: *CrystalClear*; program(s) used to solve structure: *SHELXS97* (Sheldrick, 2008 ▶); program(s) used to refine structure: *SHELXL97* (Sheldrick, 2008 ▶); molecular graphics: *XP* in *SHELXTL* (Sheldrick, 2008 ▶); software used to prepare material for publication: *SHELXTL*.

## Supplementary Material

Crystal structure: contains datablock(s) global, I. DOI: 10.1107/S1600536813006685/mw2105sup1.cif

Structure factors: contains datablock(s) I. DOI: 10.1107/S1600536813006685/mw2105Isup2.hkl

Additional supplementary materials:  crystallographic information; 3D view; checkCIF report

## Figures and Tables

**Table 1:** Hydrogen-bond geometry (Å, °)

*D*—H⋯*A*	*D*—H	H⋯*A*	*D*⋯*A*	*D*—H⋯*A*
O6—H6*A*⋯O4^i^	0.82	1.89	2.6866 (17)	165
O1*W*—H1*WA*⋯O6	0.85	1.86	2.6980 (17)	168
O1*W*—H1*WB*⋯O3^ii^	0.85	1.93	2.7237 (18)	154
O5—H5⋯O2	0.82	1.84	2.6122 (17)	156

Symmetry codes: (i) 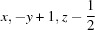; (ii) 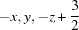.

**Fig. 1.**
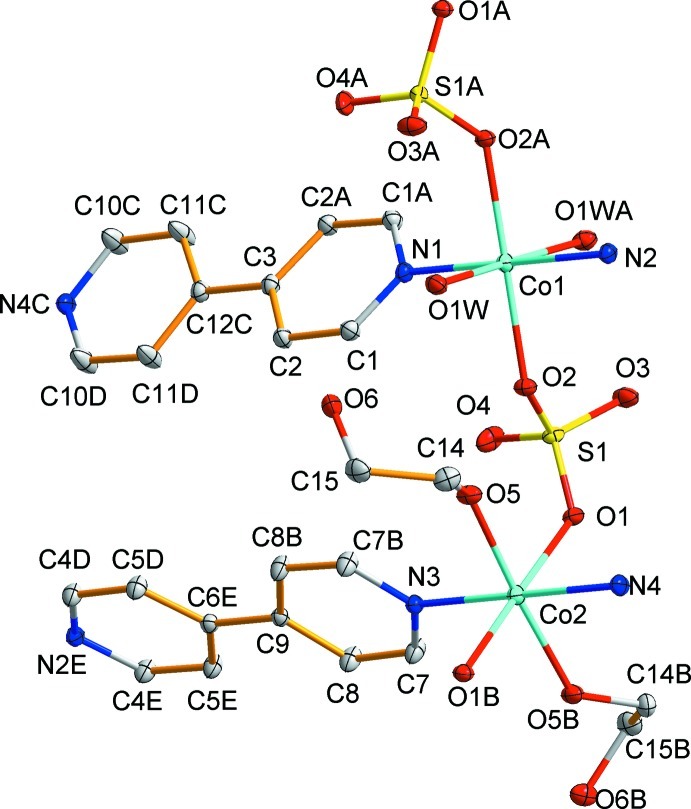
Part of the structure of the title compound, showing the atom-numbering scheme and with displacement ellipsoids drawn at the 35% probability level. H atoms have been omitted for clarity. [symmetry codes: (A) -x, y, -z + 3/2; (B) -x + 1, y, -z + 3/2; (C) x - 1/2, y + 1/2, z; (D) -x + 1/2, y + 1/2, -z + 3/2; (E) x + 1/2, y + 1/2, -z]

**Fig. 2.**
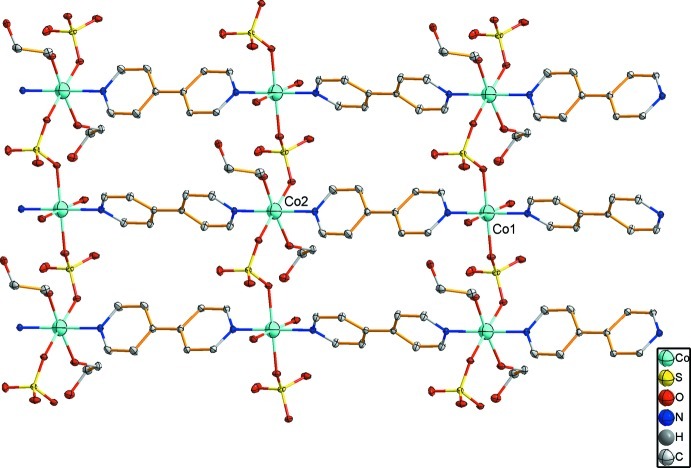
The crystal structure of the title compound, viewed along the c axis. H atoms have been omitted for clarity.

## supplementary crystallographic information

### Comment

In past decades, the design and synthesis of metal-organic coordination polymers with transition metals have attracted much attention because of their interesting topologies and potential applications in the areas of materials chemistry (Cui *et al.*, 2002; Sarma *et al.*, 2009; Zhang *et al.*, 2010). It is well known that 4,4'-bipyridine (4,4'-bipy) has been widely applied as an auxiliary bridging ligand to construct novel one-, two- and three-dimensional polymers (Tong & Chen, 2000; Croitor *et al.*, 2011). Some interesting cobalt–(4,4'-bipy) coordination polymers have been synthesized and characterized including Co(SO_4_)(C_10_H_8_N_2_)(H_2_O)_3_.2C_2_H_6_O_2_ (Lu *et al.*, 2006), [Co_2_(H_2_O)_2_(OH)_2_(4,4'-bipy)_8_](NO_3_)_2_.2(4,4'-bipy).10H_2_O (Luachan *et al.*, 2007), [Co_2_(4,4'-bipyridine)_2_(SO_4_)_2_(H_2_O)_6_].4H_2_O (Prior *et al.*, 2011), Co(SO_4_)(H_2_O)_3_(4,4'-bipy).2H_2_O and Co(Cl)_2_(DMSO)_2_(4,4'-bipy) (Lu *et al.*, 1998) and {[Co(H_2_O)_6_][Co(SO_4_)_2_(H_2_O)_2_(C_10_H_8_N_2_)][Co(SO_4_)(H_2_O)_3_(C_10_H_8_N_2_)]}_n_ (Zhong & Qian, 2012).

In the present work we describe the synthesis and structure of the new complex [Co_2_(SO_4_)_2_(C_10_H_8_N_2_)_2_(C_2_H_6_O_2_)_2_(H_2_O)_2_]_n_, (I), which displays a three-dimensional supramolecular network with two-dimensional polymeric layers and was obtained *via* a solvo-thermal reaction. It is isotypic with the previously reported Cu^II^ and Ni^II^ analogs (Zhong *et al.*, 2011; Zhong, 2013).

There are two crystallographically unique Co^II^ metal centers in the title compound, each lying on a crystallographic two-fold axis and having a slightly distorted octahedral CoN_2_O_4_ coordination environment. Atom Co1 is coordinated by two 4,4'-bipyridine N atoms (N1 and N2), two sulfate ligand O atoms (O2) and two water molecule O atoms (O1W). The second Co^II^ center (Co2) is surrounded by two N atoms (N3 and N4) from two bridging 4,4-bipyridine and four O atoms, two from bridging sulfate anions (O1) and two from ethane-1,2-diol ligands (O5) (Fig. 1). The N atoms occupy the axial positions and the O atoms the equatorial sites. The Co—N bond distances [2.1223 (19)–2.1540 (19) Å], the Co—O bond distances [2.0938 (13)-2.1262 (12) Å] and the *cis* bond angles around the Co^II^ center [87.30 (3)–92.70 (3)°] are in good accord with those found in the previously reported Co–(4,4'-bipy) complexes. The bridging 4,4'-bipyridine ligand lies on a twofold axis and links two different Co^II^ cations, giving rise to the formation of one-dimensional linear ···Co1—bipy—Co2—bipy··· chains along the *b* axis. The Co1 and Co2 centers of the adjacent ···Co1—bipy—Co2—bipy··· chains are further cross-linked by the sulfate anions in the O—S—O bridging mode, forming linear ···Co1—O—SO_2_— Co2—O—SO_2_—O··· chains running parallel to the *a* axis. The ···*M*—O—SO_2_—O—*M*··· and ···*M*—bipy—*M*··· chains are almost orthogonal resulting in a two-dimensional layered polymer (Fig. 2). In the crystal structure, the two-dimensional polymeric layers are linked by extensive O—H···O hydrogen-bonding involving the water molecules, ethane-1,2-diol molecules and sulfate anions leading to the formation of a three-dimensional supramolecular network structure.

### Experimental

Reagents and solvents used were of commercially available quality. 0.2 mmol of 4,4'-bipyridine(4,4'-bipy), 0.1 mmol of CoSO_4_.7H_2_O, 2.0 ml of ethane-1,2-diol and 1.0 ml of water were mixed and placed in a thick Pyrex tube which was sealed and heated to 413 K for 96 h. The tube was cooled to ambient temperature and pink block-shaped crystals of the title compound were obtained.

### Refinement

All non-hydrogen atoms were refined anisotropically. The aromatic H atoms were positioned geometrically and allowed to ride on their parent atoms, with C—H = 0.93 Å and *U*_iso_(H) = 1.2*U*_eq_(C). The H atoms of ethane-1,2-diol were were geometrically placed and and refined using a riding model [O—H = 0.82 Å and C—H = 0.97 Å; *U*_iso_(H) = 1.5*U*_eq_(O) and *U*_iso_(H) = 1.2*U*_eq_(C)]. The water H atoms were either located in difference Fourier maps or placed in calculated positions so as to form a reasonable hydrogen-bond network, as far as possible. Initially, their positions were refined with tight restraints on the O—H and H···H distances [0.85 (1) and 1.35 (1) Å, respectively] in order to ensure a reasonable geometry. They were then constrained to ride on their parent O atom [*U*_iso_(H) = 1.5*U*_eq_(O)].

### Crystal data

**Table:**

[Co_2_(SO_4_)_2_(C_10_H_8_N_2_)_2_(C_2_H_6_O_2_)_2_(H_2_O)_2_]	*F*(000) = 1608
*M**_r_* = 782.53	*D*_x_ = 1.707 Mg m^−^^3^
Monoclinic, *C*2/*c*	Mo *K*α radiation, λ = 0.71073 Å
Hall symbol: -C2yc	Cell parameters from 6960 reflections
*a* = 11.124 (2) Å	θ = 3.3–27.5°
*b* = 22.792 (5) Å	µ = 1.30 mm^−^^1^
*c* = 12.066 (2) Å	*T* = 223 K
β = 95.51 (3)°	Block, pink
*V* = 3045.2 (11) Å^3^	0.35 × 0.25 × 0.20 mm
*Z* = 4	

### Data collection

**Table:**

Rigaku Mercury CCD diffractometer	3453 independent reflections
Radiation source: fine-focus sealed tube	3047 reflections with *I* > 2σ(*I*)
Graphite monochromator	*R*_int_ = 0.020
Detector resolution: 28.5714 pixels mm^-1^	θ_max_ = 27.5°, θ_min_ = 3.3°
ω scans	*h* = −12→14
Absorption correction: multi-scan (*REQAB*; Jacobson, 1998)	*k* = −24→29
*T*_min_ = 0.722, *T*_max_ = 1.000	*l* = −15→15
8573 measured reflections	

### Refinement

**Table:**

Refinement on *F*^2^	Secondary atom site location: difference Fourier map
Least-squares matrix: full	Hydrogen site location: inferred from neighbouring sites
*R*[*F*^2^ > 2σ(*F*^2^)] = 0.029	H-atom parameters constrained
*wR*(*F*^2^) = 0.078	*w* = 1/[σ^2^(*F*_o_^2^) + (0.0484*P*)^2^ + 0.1682*P*] where *P* = (*F*_o_^2^ + 2*F*_c_^2^)/3
*S* = 1.05	(Δ/σ)_max_ = 0.001
3453 reflections	Δρ_max_ = 0.44 e Å^−^^3^
214 parameters	Δρ_min_ = −0.49 e Å^−^^3^
1 restraint	Extinction correction: *SHELXL97* (Sheldrick, 2008), Fc^*^=kFc[1+0.001xFc^2^λ^3^/sin(2θ)]^-1/4^
Primary atom site location: structure-invariant direct methods	Extinction coefficient: 0.0044 (3)

### Special details

**Table:**

Geometry. All e.s.d.'s (except the e.s.d. in the dihedral angle between two l.s. planes) are estimated using the full covariance matrix. The cell e.s.d.'s are taken into account individually in the estimation of e.s.d.'s in distances, angles and torsion angles; correlations between e.s.d.'s in cell parameters are only used when they are defined by crystal symmetry. An approximate (isotropic) treatment of cell e.s.d.'s is used for estimating e.s.d.'s involving l.s. planes.
Refinement. Refinement of *F*^2^ against ALL reflections. The weighted *R*-factor *wR* and goodness of fit *S* are based on *F*^2^, conventional *R*-factors *R* are based on *F*, with *F* set to zero for negative *F*^2^. The threshold expression of *F*^2^ > σ(*F*^2^) is used only for calculating *R*-factors(gt) *etc*. and is not relevant to the choice of reflections for refinement. *R*-factors based on *F*^2^ are statistically about twice as large as those based on *F*, and *R*-factors based on ALL data will be even larger.

### Fractional atomic coordinates and isotropic or equivalent isotropic displacement parameters (Å^2^)

**Table:**

	*x*	*y*	*z*	*U*_iso_*/*U*_eq_	
Co1	0.0000	0.379656 (12)	0.7500	0.01469 (10)	
Co2	0.5000	0.378829 (12)	0.7500	0.01552 (10)	
S1	0.26448 (3)	0.397753 (16)	0.89235 (3)	0.01772 (11)	
O1	0.38920 (10)	0.37753 (4)	0.88224 (9)	0.0201 (2)	
O1W	0.02400 (11)	0.37527 (4)	0.57750 (10)	0.0236 (3)	
H1WA	0.0685	0.3970	0.5413	0.035*	
H1WB	−0.0438	0.3770	0.5386	0.035*	
O2	0.18890 (10)	0.37997 (5)	0.78736 (9)	0.0200 (2)	
O3	0.21712 (11)	0.36754 (6)	0.98602 (10)	0.0294 (3)	
O4	0.26137 (11)	0.46083 (5)	0.90508 (12)	0.0374 (3)	
O5	0.34406 (10)	0.37517 (5)	0.63828 (10)	0.0242 (3)	
H5	0.2817	0.3772	0.6691	0.036*	
O6	0.19241 (10)	0.43332 (5)	0.47373 (10)	0.0309 (3)	
H6A	0.2014	0.4678	0.4567	0.046*	
N1	0.0000	0.47277 (8)	0.7500	0.0187 (4)	
N2	0.0000	0.28541 (8)	0.7500	0.0189 (4)	
N3	0.5000	0.47291 (8)	0.7500	0.0179 (4)	
N4	0.5000	0.28432 (8)	0.7500	0.0215 (4)	
C1	0.07997 (14)	0.50339 (7)	0.69724 (13)	0.0215 (3)	
H1A	0.1369	0.4829	0.6609	0.026*	
C2	0.08205 (14)	0.56396 (7)	0.69408 (13)	0.0211 (3)	
H2A	0.1381	0.5833	0.6547	0.025*	
C3	0.0000	0.59596 (10)	0.7500	0.0192 (4)	
C4	0.06167 (15)	0.25465 (7)	0.83143 (14)	0.0239 (3)	
H4A	0.1053	0.2751	0.8886	0.029*	
C5	0.06372 (15)	0.19388 (7)	0.83470 (14)	0.0234 (3)	
H5A	0.1075	0.1746	0.8933	0.028*	
C6	0.0000	0.16175 (9)	0.7500	0.0179 (4)	
C7	0.56963 (15)	0.50386 (7)	0.82523 (14)	0.0269 (4)	
H7A	0.6188	0.4834	0.8787	0.032*	
C8	0.57272 (15)	0.56448 (7)	0.82805 (14)	0.0260 (4)	
H8A	0.6234	0.5837	0.8821	0.031*	
C9	0.5000	0.59664 (9)	0.7500	0.0170 (4)	
C10	0.41224 (18)	0.25359 (8)	0.78999 (19)	0.0387 (5)	
H10A	0.3495	0.2740	0.8182	0.046*	
C11	0.40926 (17)	0.19292 (8)	0.79184 (18)	0.0388 (5)	
H11A	0.3459	0.1737	0.8214	0.047*	
C12	0.5000	0.16078 (10)	0.7500	0.0204 (4)	
C14	0.31776 (15)	0.35169 (8)	0.52895 (14)	0.0269 (4)	
H14A	0.2479	0.3261	0.5275	0.032*	
H14B	0.3857	0.3285	0.5093	0.032*	
C15	0.29304 (16)	0.40015 (8)	0.44611 (14)	0.0293 (4)	
H15A	0.3633	0.4253	0.4464	0.035*	
H15B	0.2765	0.3838	0.3720	0.035*	

### Atomic displacement parameters (Å^2^)

**Table:**

	*U*^11^	*U*^22^	*U*^33^	*U*^12^	*U*^13^	*U*^23^
Co1	0.01344 (16)	0.01259 (15)	0.01800 (17)	0.000	0.00121 (11)	0.000
Co2	0.01370 (17)	0.01303 (15)	0.01967 (17)	0.000	0.00082 (12)	0.000
S1	0.01530 (19)	0.0186 (2)	0.0190 (2)	0.00168 (14)	0.00014 (13)	−0.00353 (14)
O1	0.0145 (5)	0.0238 (6)	0.0218 (6)	0.0020 (4)	0.0012 (4)	−0.0004 (4)
O1W	0.0201 (6)	0.0301 (6)	0.0209 (6)	−0.0032 (5)	0.0029 (5)	0.0025 (4)
O2	0.0147 (5)	0.0267 (6)	0.0185 (6)	−0.0003 (4)	0.0009 (4)	−0.0023 (4)
O3	0.0198 (6)	0.0491 (8)	0.0196 (6)	−0.0006 (5)	0.0031 (5)	0.0040 (5)
O4	0.0347 (7)	0.0207 (6)	0.0548 (9)	0.0068 (5)	−0.0059 (6)	−0.0131 (6)
O5	0.0176 (6)	0.0351 (7)	0.0198 (6)	0.0006 (5)	0.0016 (5)	−0.0034 (5)
O6	0.0283 (6)	0.0273 (6)	0.0380 (7)	0.0006 (5)	0.0080 (5)	0.0119 (5)
N1	0.0192 (9)	0.0150 (8)	0.0215 (10)	0.000	0.0003 (7)	0.000
N2	0.0194 (9)	0.0147 (9)	0.0221 (10)	0.000	−0.0002 (7)	0.000
N3	0.0175 (9)	0.0147 (8)	0.0214 (10)	0.000	0.0016 (7)	0.000
N4	0.0212 (9)	0.0140 (9)	0.0295 (11)	0.000	0.0036 (8)	0.000
C1	0.0209 (7)	0.0184 (7)	0.0258 (8)	0.0009 (6)	0.0046 (6)	−0.0005 (6)
C2	0.0216 (7)	0.0176 (7)	0.0247 (8)	−0.0007 (6)	0.0058 (6)	0.0016 (6)
C3	0.0212 (11)	0.0161 (10)	0.0199 (11)	0.000	0.0000 (8)	0.000
C4	0.0271 (8)	0.0184 (7)	0.0245 (8)	0.0006 (7)	−0.0055 (7)	−0.0017 (6)
C5	0.0279 (8)	0.0182 (8)	0.0224 (8)	0.0011 (7)	−0.0063 (6)	0.0007 (6)
C6	0.0171 (10)	0.0174 (10)	0.0193 (11)	0.000	0.0024 (8)	0.000
C7	0.0312 (9)	0.0179 (8)	0.0292 (9)	0.0020 (7)	−0.0097 (7)	0.0015 (7)
C8	0.0314 (9)	0.0183 (8)	0.0258 (9)	−0.0008 (7)	−0.0099 (7)	−0.0019 (6)
C9	0.0170 (10)	0.0146 (10)	0.0198 (11)	0.000	0.0034 (8)	0.000
C10	0.0356 (10)	0.0181 (8)	0.0670 (14)	0.0013 (8)	0.0280 (10)	−0.0010 (8)
C11	0.0358 (10)	0.0178 (8)	0.0679 (14)	−0.0026 (8)	0.0314 (10)	−0.0002 (8)
C12	0.0217 (11)	0.0164 (10)	0.0229 (11)	0.000	0.0010 (9)	0.000
C14	0.0271 (8)	0.0273 (9)	0.0258 (9)	0.0018 (7)	0.0009 (7)	−0.0082 (7)
C15	0.0283 (9)	0.0386 (10)	0.0219 (9)	−0.0008 (8)	0.0072 (7)	0.0001 (7)

### Geometric parameters (Å, º)

**Table:**

Co1—O2	2.1064 (12)	C1—C2	1.381 (2)
Co1—O2^i^	2.1064 (12)	C1—H1A	0.9300
Co1—N1	2.1223 (19)	C2—C3	1.3924 (19)
Co1—O1W^i^	2.1262 (12)	C2—H2A	0.9300
Co1—O1W	2.1262 (12)	C3—C2^i^	1.392 (2)
Co1—N2	2.1480 (19)	C3—C12^iii^	1.477 (3)
Co2—O5	2.0938 (13)	C4—C5	1.386 (2)
Co2—O5^ii^	2.0938 (13)	C4—H4A	0.9300
Co2—O1	2.1081 (12)	C5—C6	1.3943 (19)
Co2—O1^ii^	2.1081 (12)	C5—H5A	0.9300
Co2—N3	2.1443 (19)	C6—C5^i^	1.3943 (19)
Co2—N4	2.1540 (19)	C6—C9^iv^	1.484 (3)
S1—O4	1.4465 (13)	C7—C8	1.382 (2)
S1—O3	1.4641 (13)	C7—H7A	0.9300
S1—O1	1.4781 (12)	C8—C9	1.390 (2)
S1—O2	1.5072 (12)	C8—H8A	0.9300
O1W—H1WA	0.8500	C9—C8^ii^	1.390 (2)
O1W—H1WB	0.8500	C9—C6^v^	1.484 (3)
O5—C14	1.428 (2)	C10—C11	1.384 (2)
O5—H5	0.8200	C10—H10A	0.9300
O6—C15	1.417 (2)	C11—C12	1.381 (2)
O6—H6A	0.8200	C11—H11A	0.9300
N1—C1^i^	1.3389 (18)	C12—C11^ii^	1.381 (2)
N1—C1	1.3389 (19)	C12—C3^vi^	1.477 (3)
N2—C4^i^	1.3409 (19)	C14—C15	1.497 (2)
N2—C4	1.3409 (19)	C14—H14A	0.9700
N3—C7	1.3366 (19)	C14—H14B	0.9700
N3—C7^ii^	1.3366 (19)	C15—H15A	0.9700
N4—C10	1.329 (2)	C15—H15B	0.9700
N4—C10^ii^	1.329 (2)		
			
O2—Co1—O2^i^	179.61 (6)	C7^ii^—N3—Co2	121.85 (10)
O2—Co1—N1	89.80 (3)	C10—N4—C10^ii^	116.4 (2)
O2^i^—Co1—N1	89.80 (3)	C10—N4—Co2	121.80 (11)
O2—Co1—O1W^i^	90.41 (5)	C10^ii^—N4—Co2	121.80 (11)
O2^i^—Co1—O1W^i^	89.60 (5)	N1—C1—C2	123.21 (15)
N1—Co1—O1W^i^	92.70 (3)	N1—C1—H1A	118.4
O2—Co1—O1W	89.60 (5)	C2—C1—H1A	118.4
O2^i^—Co1—O1W	90.41 (5)	C1—C2—C3	119.78 (15)
N1—Co1—O1W	92.70 (3)	C1—C2—H2A	120.1
O1W^i^—Co1—O1W	174.60 (6)	C3—C2—H2A	120.1
O2—Co1—N2	90.20 (3)	C2—C3—C2^i^	116.8 (2)
O2^i^—Co1—N2	90.20 (3)	C2—C3—C12^iii^	121.59 (10)
N1—Co1—N2	180.0	C2^i^—C3—C12^iii^	121.59 (10)
O1W^i^—Co1—N2	87.30 (3)	N2—C4—C5	123.32 (15)
O1W—Co1—N2	87.30 (3)	N2—C4—H4A	118.3
O5—Co2—O5^ii^	175.44 (6)	C5—C4—H4A	118.3
O5—Co2—O1	88.75 (5)	C4—C5—C6	119.89 (15)
O5^ii^—Co2—O1	91.18 (5)	C4—C5—H5A	120.1
O5—Co2—O1^ii^	91.18 (5)	C6—C5—H5A	120.1
O5^ii^—Co2—O1^ii^	88.75 (5)	C5—C6—C5^i^	116.6 (2)
O1—Co2—O1^ii^	178.39 (6)	C5—C6—C9^iv^	121.68 (10)
O5—Co2—N3	92.28 (3)	C5^i^—C6—C9^iv^	121.68 (10)
O5^ii^—Co2—N3	92.28 (3)	N3—C7—C8	123.75 (15)
O1—Co2—N3	90.81 (3)	N3—C7—H7A	118.1
O1^ii^—Co2—N3	90.81 (3)	C8—C7—H7A	118.1
O5—Co2—N4	87.72 (3)	C7—C8—C9	119.93 (15)
O5^ii^—Co2—N4	87.72 (3)	C7—C8—H8A	120.0
O1—Co2—N4	89.19 (3)	C9—C8—H8A	120.0
O1^ii^—Co2—N4	89.19 (3)	C8^ii^—C9—C8	116.4 (2)
N3—Co2—N4	180.0	C8^ii^—C9—C6^v^	121.82 (10)
O4—S1—O3	111.80 (8)	C8—C9—C6^v^	121.82 (10)
O4—S1—O1	110.55 (7)	N4—C10—C11	123.51 (17)
O3—S1—O1	109.10 (7)	N4—C10—H10A	118.2
O4—S1—O2	109.84 (7)	C11—C10—H10A	118.2
O3—S1—O2	108.02 (7)	C12—C11—C10	120.31 (17)
O1—S1—O2	107.40 (7)	C12—C11—H11A	119.8
S1—O1—Co2	132.83 (7)	C10—C11—H11A	119.8
Co1—O1W—H1WA	127.6	C11^ii^—C12—C11	116.0 (2)
Co1—O1W—H1WB	110.5	C11^ii^—C12—C3^vi^	122.02 (11)
H1WA—O1W—H1WB	102.6	C11—C12—C3^vi^	122.02 (11)
S1—O2—Co1	130.19 (7)	O5—C14—C15	110.40 (14)
C14—O5—Co2	133.90 (10)	O5—C14—H14A	109.6
C14—O5—H5	109.5	C15—C14—H14A	109.6
Co2—O5—H5	113.0	O5—C14—H14B	109.6
C15—O6—H6A	109.5	C15—C14—H14B	109.6
C1^i^—N1—C1	117.17 (19)	H14A—C14—H14B	108.1
C1^i^—N1—Co1	121.41 (10)	O6—C15—C14	109.59 (14)
C1—N1—Co1	121.41 (10)	O6—C15—H15A	109.8
C4^i^—N2—C4	116.96 (19)	C14—C15—H15A	109.8
C4^i^—N2—Co1	121.52 (10)	O6—C15—H15B	109.8
C4—N2—Co1	121.52 (10)	C14—C15—H15B	109.8
C7—N3—C7^ii^	116.29 (19)	H15A—C15—H15B	108.2
C7—N3—Co2	121.85 (10)		
			
O4—S1—O1—Co2	−77.34 (11)	O1—Co2—N3—C7	78.37 (10)
O3—S1—O1—Co2	159.32 (8)	O1^ii^—Co2—N3—C7	−101.63 (10)
O2—S1—O1—Co2	42.47 (10)	O5—Co2—N3—C7^ii^	−12.85 (10)
O5—Co2—O1—S1	−28.26 (9)	O5^ii^—Co2—N3—C7^ii^	167.15 (10)
O5^ii^—Co2—O1—S1	156.30 (9)	O1—Co2—N3—C7^ii^	−101.63 (10)
N3—Co2—O1—S1	64.00 (8)	O1^ii^—Co2—N3—C7^ii^	78.37 (10)
N4—Co2—O1—S1	−116.00 (8)	O5—Co2—N4—C10	−65.05 (12)
O4—S1—O2—Co1	−71.63 (10)	O5^ii^—Co2—N4—C10	114.95 (12)
O3—S1—O2—Co1	50.55 (10)	O1—Co2—N4—C10	23.74 (12)
O1—S1—O2—Co1	168.10 (7)	O1^ii^—Co2—N4—C10	−156.26 (12)
N1—Co1—O2—S1	69.57 (8)	O5—Co2—N4—C10^ii^	114.95 (12)
O1W^i^—Co1—O2—S1	−23.13 (9)	O5^ii^—Co2—N4—C10^ii^	−65.05 (12)
O1W—Co1—O2—S1	162.27 (8)	O1—Co2—N4—C10^ii^	−156.26 (12)
N2—Co1—O2—S1	−110.43 (8)	O1^ii^—Co2—N4—C10^ii^	23.74 (12)
O1—Co2—O5—C14	−150.40 (14)	C1^i^—N1—C1—C2	−0.83 (11)
O1^ii^—Co2—O5—C14	27.99 (14)	Co1—N1—C1—C2	179.17 (11)
N3—Co2—O5—C14	118.84 (14)	N1—C1—C2—C3	1.6 (2)
N4—Co2—O5—C14	−61.16 (14)	C1—C2—C3—C2^i^	−0.77 (10)
O2—Co1—N1—C1^i^	−134.02 (9)	C1—C2—C3—C12^iii^	179.23 (10)
O2^i^—Co1—N1—C1^i^	45.98 (9)	C4^i^—N2—C4—C5	−0.20 (12)
O1W^i^—Co1—N1—C1^i^	−43.61 (9)	Co1—N2—C4—C5	179.80 (12)
O1W—Co1—N1—C1^i^	136.39 (9)	N2—C4—C5—C6	0.4 (2)
O2—Co1—N1—C1	45.98 (9)	C4—C5—C6—C5^i^	−0.19 (11)
O2^i^—Co1—N1—C1	−134.02 (9)	C4—C5—C6—C9^iv^	179.81 (11)
O1W^i^—Co1—N1—C1	136.39 (9)	C7^ii^—N3—C7—C8	−0.21 (13)
O1W—Co1—N1—C1	−43.61 (9)	Co2—N3—C7—C8	179.79 (13)
O2—Co1—N2—C4^i^	−133.47 (9)	N3—C7—C8—C9	0.4 (3)
O2^i^—Co1—N2—C4^i^	46.53 (9)	C7—C8—C9—C8^ii^	−0.19 (12)
O1W^i^—Co1—N2—C4^i^	136.13 (9)	C7—C8—C9—C6^v^	179.81 (12)
O1W—Co1—N2—C4^i^	−43.87 (9)	C10^ii^—N4—C10—C11	0.26 (17)
O2—Co1—N2—C4	46.53 (9)	Co2—N4—C10—C11	−179.74 (17)
O2^i^—Co1—N2—C4	−133.47 (9)	N4—C10—C11—C12	−0.5 (3)
O1W^i^—Co1—N2—C4	−43.87 (9)	C10—C11—C12—C11^ii^	0.24 (15)
O1W—Co1—N2—C4	136.13 (9)	C10—C11—C12—C3^vi^	−179.76 (15)
O5—Co2—N3—C7	167.15 (10)	Co2—O5—C14—C15	−111.40 (14)
O5^ii^—Co2—N3—C7	−12.85 (10)	O5—C14—C15—O6	−60.27 (18)

Symmetry codes: (i) −*x*, *y*, −*z*+3/2; (ii) −*x*+1, *y*, −*z*+3/2; (iii) *x*−1/2, *y*+1/2, *z*; (iv) *x*−1/2, *y*−1/2, *z*; (v) *x*+1/2, *y*+1/2, *z*; (vi) *x*+1/2, *y*−1/2, *z*.

### Hydrogen-bond geometry (Å, º)

**Table:**

*D*—H···*A*	*D*—H	H···*A*	*D*···*A*	*D*—H···*A*
O6—H6*A*···O4^vii^	0.82	1.89	2.6866 (17)	165
O1*W*—H1*WA*···O6	0.85	1.86	2.6980 (17)	168
O1*W*—H1*WB*···O3^i^	0.85	1.93	2.7237 (18)	154
O5—H5···O2	0.82	1.84	2.6122 (17)	156

Symmetry codes: (i) −*x*, *y*, −*z*+3/2; (vii) *x*, −*y*+1, *z*−1/2.
